# Vertical distribution of prokaryotes communities and predicted metabolic pathways in New Zealand wetlands, and potential for environmental DNA indicators of wetland condition

**DOI:** 10.1371/journal.pone.0243363

**Published:** 2021-01-06

**Authors:** Jamie R. Wood, Olivia R. Burge, Nic Bolstridge, Karen Bonner, Beverley Clarkson, Theresa L. Cole, Carina Davis, Alex Fergus, Perēri King, Michelle M. McKeown, Chris Morse, Sarah J. Richardson, Hugh Robertson, Janet M. Wilmshurst

**Affiliations:** 1 Manaaki Whenua–Landcare Research, Lincoln, New Zealand; 2 Manaaki Whenua–Landcare Research, Hamilton, New Zealand; 3 Department of Biology, Ecology and Evolution, University of Copenhagen, Copenhagen, Denmark; 4 Maungaharuru-Tangitū Trust, Hawke's Bay Mail Centre, Napier, New Zealand; 5 Department of Conservation, Nelson, New Zealand; 6 School of Environment, The University of Auckland, Auckland, New Zealand; University of Hyogo, JAPAN

## Abstract

Globally, wetlands are in decline due to anthropogenic modification and climate change. Knowledge about the spatial distribution of biodiversity and biological processes within wetlands provides essential baseline data for predicting and mitigating the effects of present and future environmental change on these critical ecosystems. To explore the potential for environmental DNA (eDNA) to provide such insights, we used 16S rRNA metabarcoding to characterise prokaryote communities and predict the distribution of prokaryote metabolic pathways in peats and sediments up to 4m below the surface across seven New Zealand wetlands. Our results reveal distinct vertical structuring of prokaryote communities and metabolic pathways in these wetlands. We also find evidence for differences in the relative abundance of certain metabolic pathways that may correspond to the degree of anthropogenic modification the wetlands have experienced. These patterns, specifically those for pathways related to aerobic respiration and the carbon cycle, can be explained predominantly by the expected effects of wetland drainage. Our study demonstrates that eDNA has the potential to be an important new tool for the assessment and monitoring of wetland health.

## Introduction

Wetlands are globally important ecosystems for their biodiversity, cultural, and recreational values, and regulatory and functional roles [1–3] yet are among the most threatened by anthropogenic modification and global climate change. Approximately 4–6% of Earth’s land surface area is classified as wetland [1], but since 1900 CE 64–71% of global wetlands have been lost through drainage, infilling or agricultural conversion [4]. Moreover, increasing atmospheric carbon dioxide (CO_2_) concentrations and concomitant global warming have the potential to significantly influence biological communities, ecological functioning and processes in wetlands [5, 6], with the potential for feedback loops via increased wetland greenhouse gas emissions under a warming world [7, 8]. Anthropogenic modification of wetlands, particularly drainage, can lead to further increases in CO2 emissions [9]. An understanding of how biodiversity and biological processes are distributed within wetlands is therefore essential for predicting how present and future environmental change might affect these critical ecosystems.

Prokaryotes (archaea and bacteria) represent a large portion of the total biodiversity in wetlands [10]. Moreover, prokaryotes play a major role in providing wetland services and driving function through facilitating key biogeochemical processes that contribute to nutrient cycling, productivity and greenhouse gas cycles [11]. Over the past two decades, DNA metabarcoding (specifically using the V4 region of the 16S rRNA gene) has allowed wetland prokaryote communities (including unculturable taxa) to be studied in more detail than was previously possible. Such work has demonstrated that at broad taxonomic ranks (e.g. phyla), wetland prokaryote communities are strongly structured by hydrological, chemical, physical and biological (i.e. vegetation) characteristics [12], and these patterns appear remarkably consistent between landmasses irrespective of geographic distance. Prokaryote communities respond rapidly to changes in these characteristics [13], whether caused by natural processes or anthropogenic activities. Accordingly, patterns of prokaryote community composition in wetland peats and sediments (hereafter wetland soils) may provide insights into functional differences between distinct wetland types or may serve as useful indicators of anthropogenic impacts on wetlands [12–16]; and moreover could provide useful indicators for assessing wetland condition and monitoring wetland restoration.

In addition to the variability observed between different wetland types and states of modification, it is well-established that prokaryote communities change vertically within an individual wetland in response to water saturation and oxygen levels. Key wetland processes (such as fermentation and methanogenesis) occur at depth and anthropogenic modifications to the surface of wetlands may have repercussions at depth through lowering of the water table and increasing oxygen levels [17]. Therefore, considering vertical profiles is a necessary aspect of wetland microbial studies. Despite this, soil depth has so far been explored as a variable in only a relatively small proportion of wetland prokaryote studies, with substantial variation in the depth ranges examined by these studies. Perhaps reflecting difficulties in sampling at depth, most have focussed on relatively shallow depth ranges (< 65 cm; e.g. [18–20]), with just a few studies extending to depths of 1–7 m below the surface (e.g. [16, 21, 22]).

Wetlands represented approximately 10% of New Zealand’s total land area at the time of initial human settlement (13^th^ Century AD) (~ 2.5 million ha), but fires, reclamation, nutrient runoff from agricultural land and drainage have since contributed to extensive (~ 90%) loss of natural wetlands [23, 24]. Calculated ongoing regional rates of wetland area loss in New Zealand are equivalent to global averages (0.5% yr^-1^) [4, 25]. In this study we use 16S rRNA metabarcoding to characterise the vertical stratification of prokaryote communities in wetland soils between 1 and 4 m below the surface at seven New Zealand wetlands. Further, we use predicted metabolic pathways from three wetlands of the same physical and vegetation characteristics (restiad ombrotrophic bogs) that span a condition gradient to explore whether prokaryote driven processes occurring at depth might be impacted by anthropogenic modification of wetlands. Our study also helps overcome a strong geographic bias in wetland prokaryote studies towards wetlands from Europe, Asia and the Americas, and provides new data on prokaryote community composition within Southern Hemisphere wetlands.

## Materials and methods

### Study sites and sampling

We collected soil cores from seven different wetlands in New Zealand (S1 Fig). Within our sampling design we incorporated both a variety of different wetland types (ombrotrophic bogs, valley-floor fen, a coastal spring-fed wetland and an infilled calcareous lagoon) and replicates of a single wetland type (ombrotrophic bogs) across a degradation spectrum, as defined by wetland condition index scores between 0 (most degraded) and 25 (intact) (S2 Fig). A wide range of factors are considered in the calculation of the wetland condition index, including hydrological integrity, physio-chemical parameters, ecosystem intactness, browsing, predation and harvesting regimes and dominance of native plants (see [26] for full description of methods). Cores were taken at the centre of 10 x 10 m plots, in which vegetation was assessed following Hurst & Allen [27]. Measurements of surface pH, conductivity and depth to the water table were also recorded. Two wetlands (Glendhu and Hinekatorangi) transitioned from peat to mainly non-peat sediment at ~40 cm and 85 cm depths respectively. At these sites additional pH measurements were recorded at depth (205 cm and 112 cm respectively) and were applied to samples taken from these different substrate types. Plant nomenclature follows the New Zealand Plant Conservation Network (https://www.nzpcn.org.nz/; May 2020). Details of the sampled wetlands and cores are presented in Table 1, with core stratigraphies and locations in S1 Fig.

**Table 1:** Characteristics and descriptions of study sites.

Site name	Core id	Maximum core depth (cm)	Latitude/ Longitude	Site type	Wetland condition (ombrotrophic bogs only)	Current extent (ha)	pH	Water table depth at time of sampling (cm)	Conductivity (uS)	Dominant vegetation cover and notes
Awarua	X17/1	138	46.5102° S, 168.7160° E	Ombotrophic bog	21.67	12,000	3.77	30	194.5	*Empodisma minus* (Restionaceae) and *Gleichenia dicarpa* (Gleichenaceae), with occasional *Leptospermum scoparium* and *Machaerina tenax*.
Bayswater	X17/2	200	46.1345° S, 168.0627° E	Ombotrophic bog	19.05	520	4.16	18	88.3	*Empodisma minus* and *Gleichenia dicarpa*. Drainage exists around wetland margins and exotic tree encroachment was occurring near to the core site.
Dunearn	X17/3	200	46.0011° S, 168.1963° E	Ombotrophic bog	15.25	60	4.03	28	71.6	*Empodisma minus* (~40% dead plants), with some bare peat patches, lichens and exotic *Rumex acetosella*. Drainage has occurred over many years and the peat surface is intensely hummocked.
Eweburn1	X18/8	120	45.3325° S, 167.8058° E	Bog		0.75	4.32	7	59.2	*Empodisma minus*, *Dracophyllum oliveri* and *Sphagnum* mosses. Gravel ridges along the margins of the wetland contained *Leptospermum scoparium* and *Halocarpus bidwillii*.
Eweburn2	X18/9	100	45.3350° S, 167.8097° E	Valley-floor fen		220	6.39	8	47.5	Mosses and *Carex* sedges, with burnt trees (*Leptospermum scoparium*). Situated within the Dome Mire/Dismal Swamp wetland complex [54].
Hinekatorangi	X17/17	300	39.3667° S, 176.8942° E	Coastal spring-fed wetland		3	6.82 (peat); 7.39 (silt)	Not measured	Not measured	Relatively dry part of wetland dominated by *Juncus* rushes. Preserved gastropod shells within the deeper silts.
Glendhu	X18/89	400	44.6639° S, 169.0450° E	Infilled alkaline lagoon		12.5	6.75 (peat); 7.44 (silt)	Not measured	Not measured	Grassed wetland ~10 m from edge of *Typha orientalis* stand at eastern margin of Glendhu. Dark organic peat at least 2.9 m thick recorded near the core site by Trotter [56]. Gastropod shells and extinct bird bones [55] within deeper calcareous silts.

Cores were stored frozen at Manaaki Whenua Landcare Research, Lincoln, New Zealand, until subsampling was undertaken. The subsampling procedure involved partly thawing the core at room temperature, removing the surface of the core by scraping with a sterile scalpel blade, then UV irradiating the freshly exposed surface for 30 minutes to reduce potential contamination. Samples were then cut from the core using sterile scalpel blades. All subsampling was performed within a still-air benchtop sampling hood, designed following Wood and Wilmshurst [28] but widened to accommodate 50 cm long cores. The sampling hood was cleaned between cores using a 10% Decon 90^TM^ solution, 10% sodium hypochlorite solution and UV light (minimum 30 minutes). Subsampling depth intervals for each core are shown in S1 Fig.

### DNA extraction and 16S rRNA library construction

DNA extractions and barcoding PCR setups were performed in an ultra-clean laboratory at Manaaki Whenua Landcare Research, Lincoln, New Zealand. PCR, indexing PCR setup and downstream procedures were performed in a standard molecular laboratory at the same facility. DNA was extracted from peat samples (0.4–0.89 g; mean = 0.47 g) from the Awarua, Bayswater and Dunearn cores using a Qiagen Dneasy PowerSoil Kit, with bead beating undertaken using a vortex adapter. DNA was extracted from peat/sediment samples (0.46–3.46 g; mean = 1.66) from Glendhu, Hinekatorangi, Eweburn 1 and Eweburn 2 cores using a Qiagen Dneasy PowerMax Soil kit, with bead beating undertaken using an Omni Bead Ruptor set at 3.1ms^-1^ for 30 sec. The solution C3 incubation step was performed at 4°C for ~20 hours (overnight).

Metabarcoding amplicons were generated using primers 515F [29] and 806R [30] for the V4 region of the 16S rRNA gene with Illumina linker sequences added to the 5’ ends (i5: TCGTCGGCAGCGTC; i7: GTCTCGTGGGCTCGG). These PCRs were performed in 12.5 μl volumes containing 1 μg/mL BSA, 1x PCR buffer, 2 mM MgSO_4_, 10 μM each dNTP, 0.4 μM each primer, 1U Platinum HiFi Taq (Invitrogen) and 1 μL of eluted DNA. Thermocycling conditions were as follows: 94°C for 2 min; 40 cycles of 94°C for 30 sec, 50°C for 30 sec and 68°C for 40 sec; with a final step of 68°C for 10 min.

Sample-specific dual indexed sequencing adapters were added in a second-round of PCR (20 μL volumes) consisting of 0.25 μL of iTaq DNA polymerase (Intron) and the manufacturer’s reaction mix, 0.75 μL of each adapter (2 μM working stocks) and 1 μL of amplified library. Indexing PCRs were cycled at 94°C for 2 min; 7 cycles of 94°C for 20 sec, 55°C for 10 sec and 72°C for 30 sec; with a final step of 72°C for 10 min. Low yield libraries were repeated with additional cycles. Quantity and fragment size distribution of indexed libraries were checked using a LabChip (PerkinElmer), and these were then pooled in equal amounts for sequencing.

### Sequencing and bioinformatics

The combined libraries were sequenced using a 250 bp paired-end kit on an Illumina MiSeq at Macrogen (South Korea) (Awarua, Bayswater, Dunearn) and Auckland University (New Zealand) (Eweburn, Hinekatorangi, Glendhu). Demultiplexed reads were processed using QIIME2 and associated plugins [31]. Denoising was performed following the DADA2 pipeline [32]. Taxonomic classification was performed using QIIME2’s Naive Bayes classifier trained on the Greengenes 13_8 99% operational taxonomic units (OTUs) (trimmed to the V4 region flanked by the 515F/806R primers) [33]. Taxa identified as present in blank controls (S1 Table) were filtered from the sample data, except where they were > 10 times more abundant in a sample compared to a control or were present in > 10 times as many samples as controls. OTUs present in < 3 samples were filtered from the dataset, as were low diversity samples (those with < 20 OTUs). Richness and diversity indices (Faith’s Phylogenetic Diversity and Shannon Diversity) were calculated using QIIME2. We used linear mixed-effects models to determine the effect of pH on richness and each of the two diversity metrics. We fitted a quadratic model and compared this to a linear model, and an intecept-only (null) model without a term for pH. All models included sample depth as a covariate. In all instances, the quadratic model had the lowest AIC value relative to the linear and intercept-only models (delta AIC was always >5). We used the effects package [34] to obtain fitted values for each quadratic model and used these to plot relationships with 95% confidence intervals. Model fit was assessed using the coefficient of determination (*r*^2^).The relative abundance of MetaCyc [35] metabolic pathways were predicted for each sample using PICRUSt2 [36]; a tool that provides metagenome predictions based on prokaryote 16S rRNA amplicon sequencing data. The PICRUSt approach provides a tool for cost-effective scanning for functional patterns across large sample size 16S rRNA metabarcoding datasets, though as a predictive method it has some known limitations compared with shotgun metagenomic data [36]. We compared the composition of the metabolic pathways visually, across sites and depth, using non-metric multidimensional scaling ordination (NMDS) in R version 3.6.0 [37]. We transformed the data by taking a log base 2 transformation for values greater than 0, and the alternative Gower distance as proposed by Anderson et al. [38]. To assess changes in predicted metabolic pathway composition related to depth, we clustered samples using the Ward’s criterion hierarchical clustering with the log transformation and alternative Gower distance described above, and cut the dendrogram at k = 7, which would have allowed our sites (n = 7) to fall into entirely separate clusters, if strong between-site compositional differences occurred. We analysed how the resulting clusters varied with depth in each site. Indicator value scores were calculated using the R labdsv package [39] and function indval, reflecting a combination of relative abundance within a cluster and fidelity to that cluster.

## Results

### Richness and diversity

**Fig 1:**
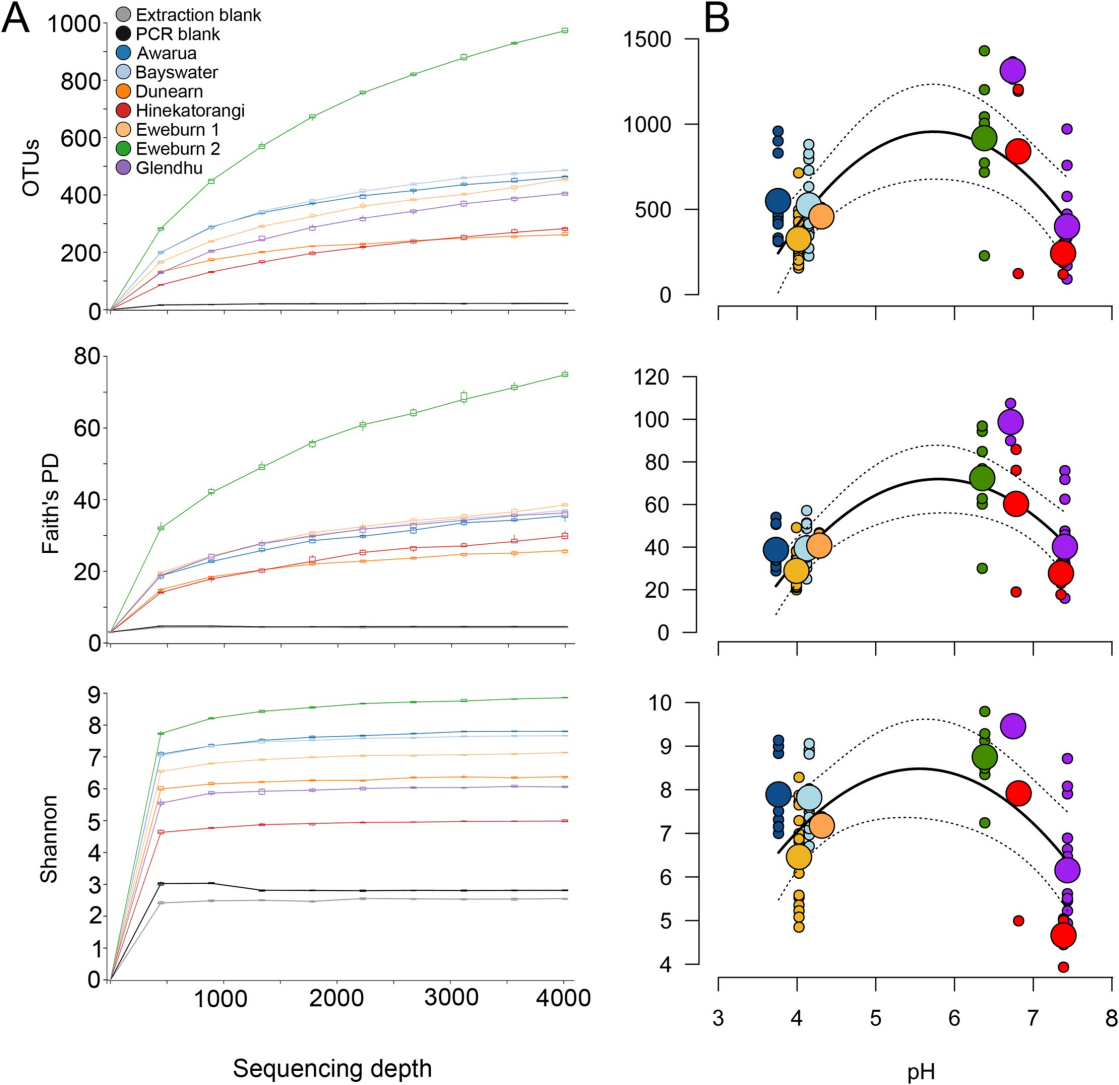
Richness and diversity of prokaryote 16S rRNA communities in New Zealand wetlands. a) Rarefied richness and diversity curves; b) range of richness and diversity values for individual samples from each core (small circles) plotted against pH. Large circles represent mean values for each site or distinct soil/sediment unit within a site. Solid lines represent fitted quadratic curves with dashed lines representing the lower and upper bounds of 95% confidence intervals. Note that values in B are based on a sequencing depth of 4000 reads.

The rarefaction curves for relative richness (number of observed OTUs), Faith’s phylogenetic diversity and Shannon diversity were relatively consistent between wetlands (Fig 1), except for Eweburn 2 which exhibited notably greater values for each metric (Fig 1). Among wetland types, bogs tended to have greater OTU richness and diversity than the other wetland types, except for Dunearn, which had the lowest richness and phylogenetic diversity values. Quadratic relationships had strong statistical support relative to either linear, or intercept-only models (see Methods), suggesting that intermediate levels of pH promote the highest microbial richness and diversity across wetlands (OTU model *r*^2^ = 0.63; Faith’s PD model *r*^2^ = 0.70; Shannon model *r*^2^ = 0.81). Although prokaryote reads were obtained from the extraction blanks and PCR blanks, the blanks were characterised by very low observed OTUs, phylogenetic diversity and Shannon diversity compared to the wetland soil samples (Fig 1).

### Community composition

**Fig 2:**
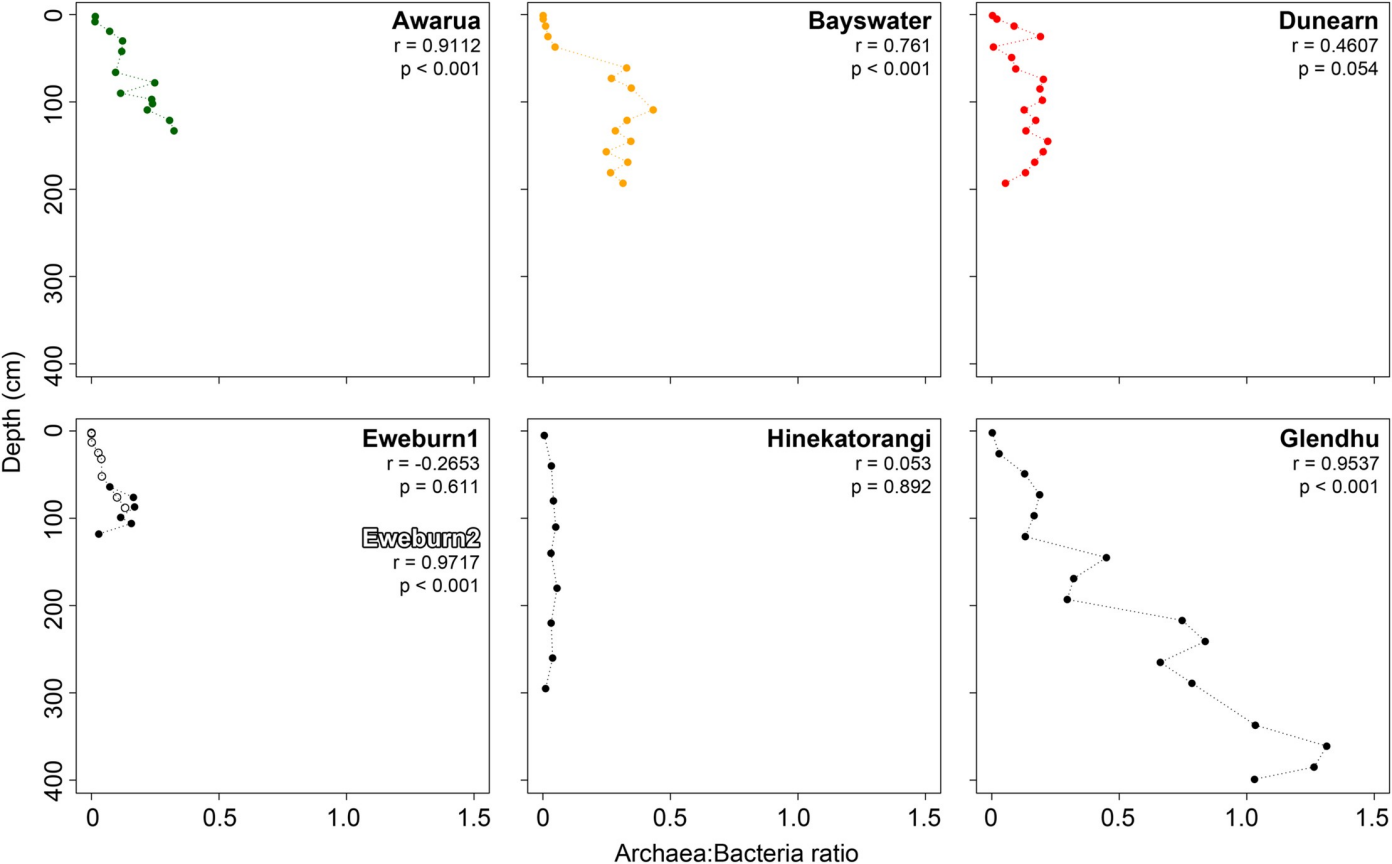
Relationship between Archaea: Bacteria ratio (assigned reads) and depth within seven New Zealand wetlands. For ombrotrophic bogs, colours reflect wetland condition: Red, Dunearn (most modified condition); Orange, Bayswater (moderate condition); Green, Awarua (most intact condition).

Archaea were rare or absent in the surface layers of all wetlands but increased with depth. Overall, there was a positive correlation between archaea:bacteria ratios and depth (Pearson’s correlation coefficient r = 0.779, p>0.0001). However, the strength and significance of correlations between these two variables differed between sites (Fig 2). Significant (p<0.001) correlations were seen at Awarua, Bayswater, Eweburn2 and Glendhu, with the strongest positive r values (>0.9) at Eweburn2, Glendhu and Awarua (Fig 2). Within the three ombrotrophic bog sites both the strength (r value) and significance of the correlations declined with decreasing wetland condition index. The two longest cores, Hinekatorangi and Glendhu, exhibited quite different trends in archaea:bacteria ratios. At Hinekatorangi, prokaryote communities were dominated by bacteria and there was little increase in the relative proportion of archaea with changing depth (Fig 2). By contrast, at Glendhu the ratio of archaea:bacteria increased consistently with depth until archaeal reads numerically dominated bacterial reads between 3 and 4m depth (Fig 2).

**Fig 3:**
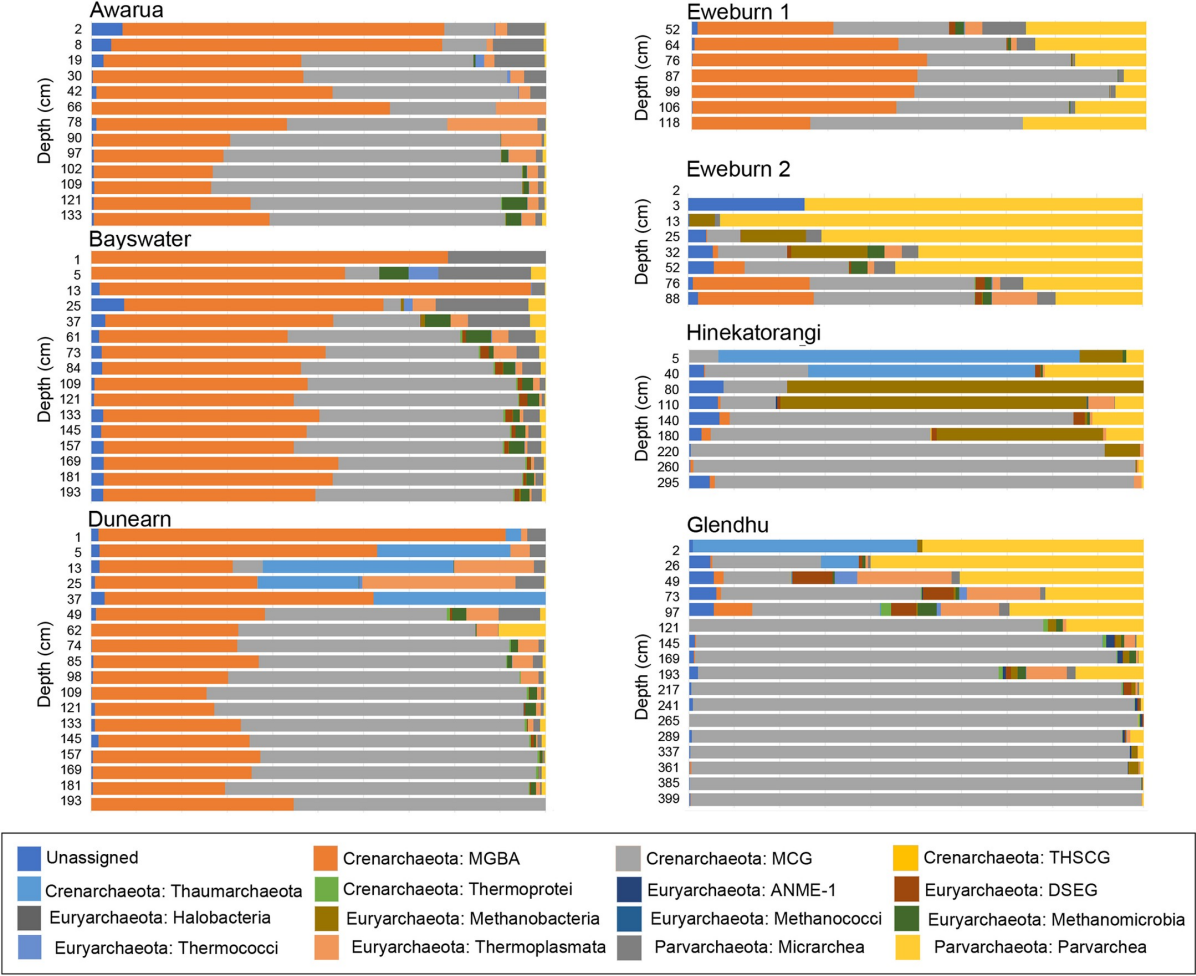
Archaea community composition at the taxonomic level of class.

Archaeal community composition was assessed at the level of class, as this was the lowest taxonomic rank to which most archaeal reads were successfully classified. Archaeal communities varied between localities (Fig 3). Communities in the three ombrotrophic bogs (Awarua, Bayswater and Dunearn) had high proportions of the Crenarchaeota classes MBGA and MCG, which together represented >50% of archaea reads in all but two samples (Fig 3). Three of the other wetlands (Eweburn 2, Hinekatorangi and Glendhu) exhibited relatively high proportions of MCG and the Parvarchaeota class Parvarchaea (Fig 3). In these wetlands Parvarchaea and MCG had differing responses to increasing depth, decreasing and increasing in relative abundance respectively (Fig 3). Archaea communities in Eweburn 1 appeared to be intermediate between these two groups, with high proportions of MBGA and MCG like the ombrotrophic bogs, and proportions of Parvarchaea comparable to those seen in Eweburn 2, Hinekatorangi and Glendhu (Fig 3).

**Fig 4:**
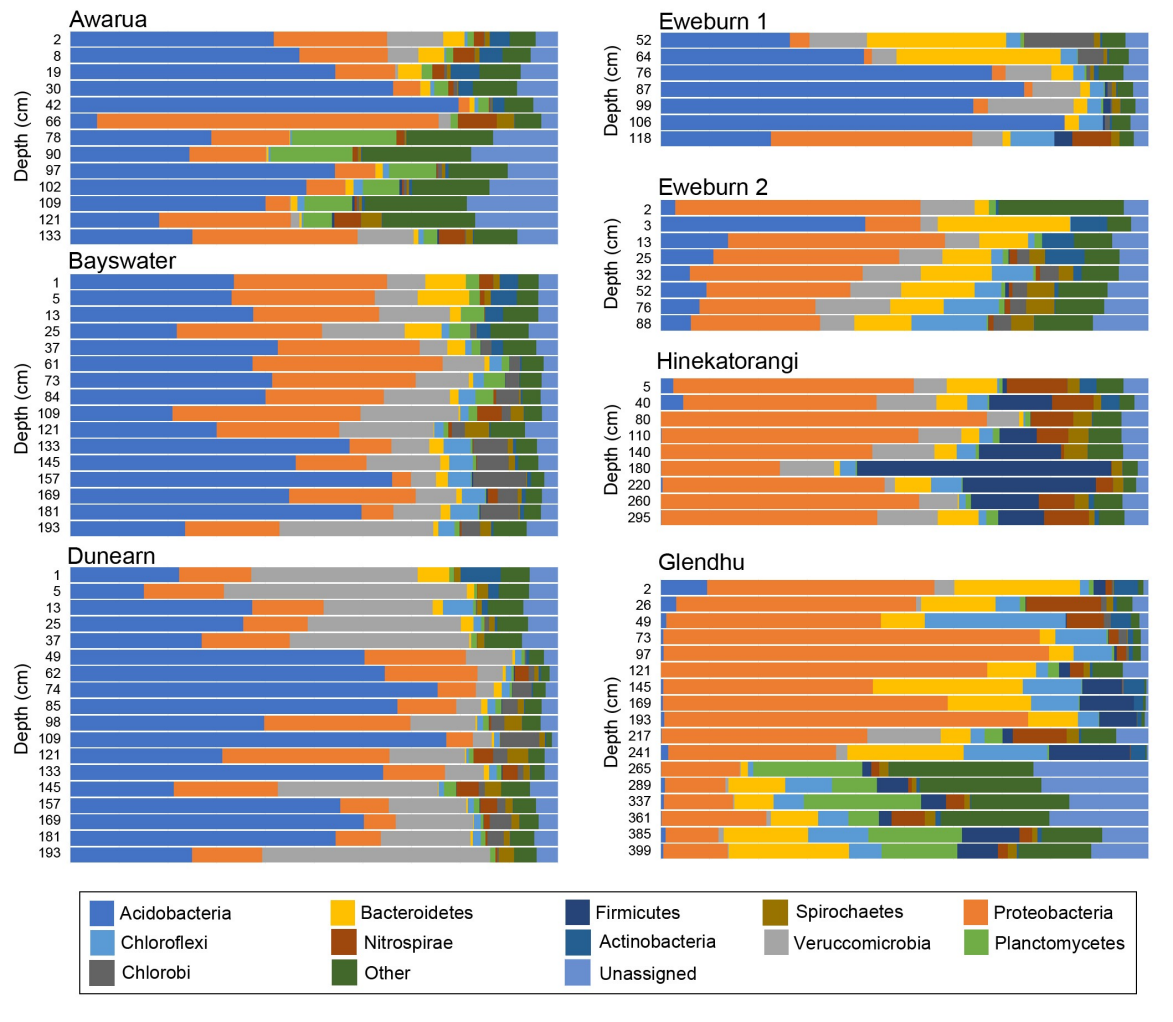
Bacteria community composition at the taxonomic level of phyla.

Due to the large number of bacterial taxa resolved, bacterial communities were assessed at the level of phyla. Acidobacteria dominated bacterial communities in the ombrotrophic bogs (Awarua, Bayswater, Dunearn) and Eweburn 1; sites characterised by their low pH values (< 4.5). There was no clear trend in the relative abundance of Acidobacteria with depth (Fig 4). By contrast, Acidobacteria were a relatively minor component of the bacterial communities at the highest pH sites, Hinekatorangi and Glendhu, where they occurred mainly at low abundance within surface peats and were rare in deeper silts (S1 Fig; Fig 4).

Proteobacteria were also a major component of the wetland bacterial communities. Proteobacteria was the most abundant phyla at Hinekatorangi and Glendhu but was also relatively common at other sites (Fig 4). Other phyla that occurred in notable abundances included Verrucomicrobia, Bacteroidetes, Chloroflexi, Planctomycetes, Firmicutes, Nitrospirae, Chlorobi, Spirochaetes and Actinobacteria (Fig 4). Some of these phyla appeared to exhibit patterns of relative abundance related to depth. For example, Planctomycetes occurred more commonly at greater depths in Awarua and Glendhu (Fig 4). However, these patterns were not always consistent between sites. For example, Chlorobi declined with depth in Eweburn 1 but increased with depth in Dunearn, Bayswater and Eweburn 2 (Fig 4). Within the ombrotrophic bogs, the relative abundance of Veruccomicrobia increased with declining wetland condition score (Fig 4).

**Fig 5:**
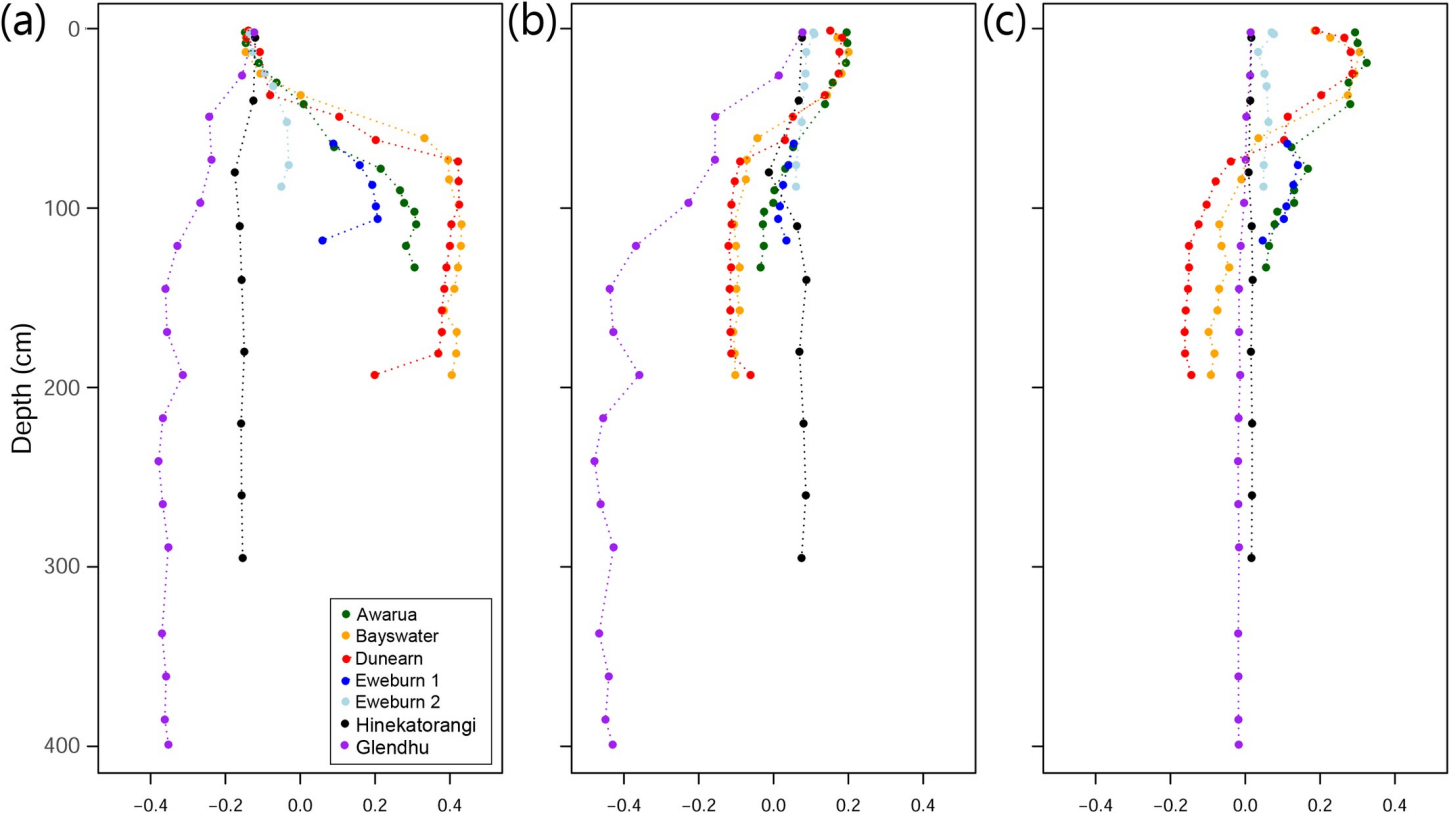
Bray-Curtis PCA (total prokaryote community composition) scores plotted against depth. a) axis 1 (15.14% variation explained); b) axis 2 (8.77% variation explained); c) axis 3 (6.48% variation explained).

Bray-Curtis dissimilarity PCA axis 1 scores for Archaea and Bacteria combined at all sites were similar at the surface but diverged with depth (Fig 5). The surface samples separated along axes 2 and 3 (Fig 5).

### Predicted metabolic pathways

The PICRUSt2 analysis predicted the presence of 6,760 functional gene orthologs, 2,115 enzymes and 410 metabolic pathways across all wetland samples.

**Fig 6:**
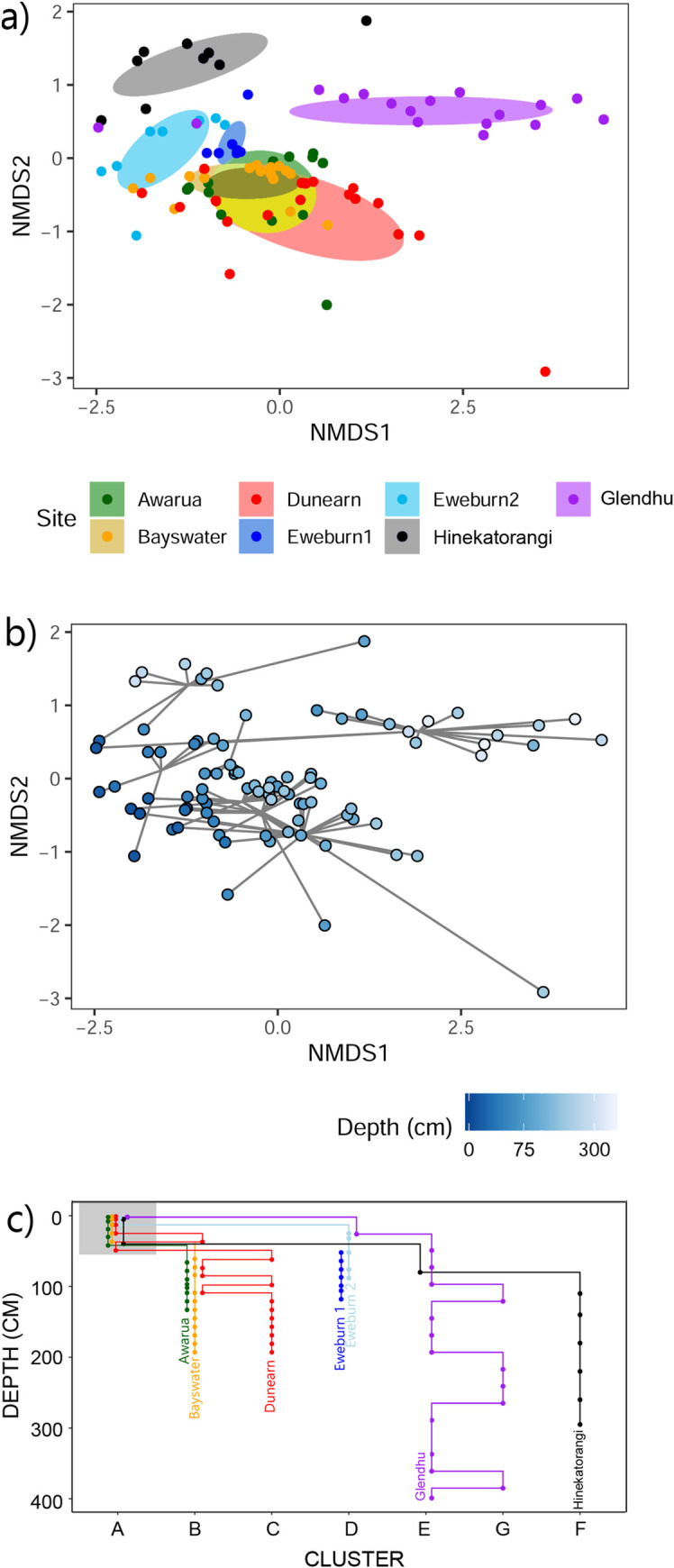
NMDS ordination of samples based on metabolic pathway composition. Coloured by a) locality and b) depth. c) Clusters assigned using hierarchical cluster analysis (cut at k = 7) plotted against depth.

In the NMDS the hulls of the three acidic ombrotrophic bogs overlapped, while other localities formed distinct hulls (Fig 6A). However, there was also a pattern related to depth, where surface samples from each locality plotted relatively close together, and deeper samples became more dispersed (Fig 6B).

Samples were assigned to seven clusters based on the composition of predicted metabolic pathways (S3 Fig). As seen in the NMDS, surface samples from all wetlands fell into the same cluster (A), with differentiation of wetland sites occurring mostly at depths below ~50 cm (Fig 6C). By 100 cm depth most wetland sites had segregated into distinct clusters as follows: Cluster B, Awarua and Bayswater; Cluster C, Dunearn; Cluster D, Eweburn 1 and Eweburn 2; Cluster E and G, Glendhu; Cluster F, Hinekatorangi (Fig 6C). The metabolic pathways with the highest indicator value scores in each cluster are shown in S2 Table.

### Effects of anthropogenic modification on function

**Fig 7:**
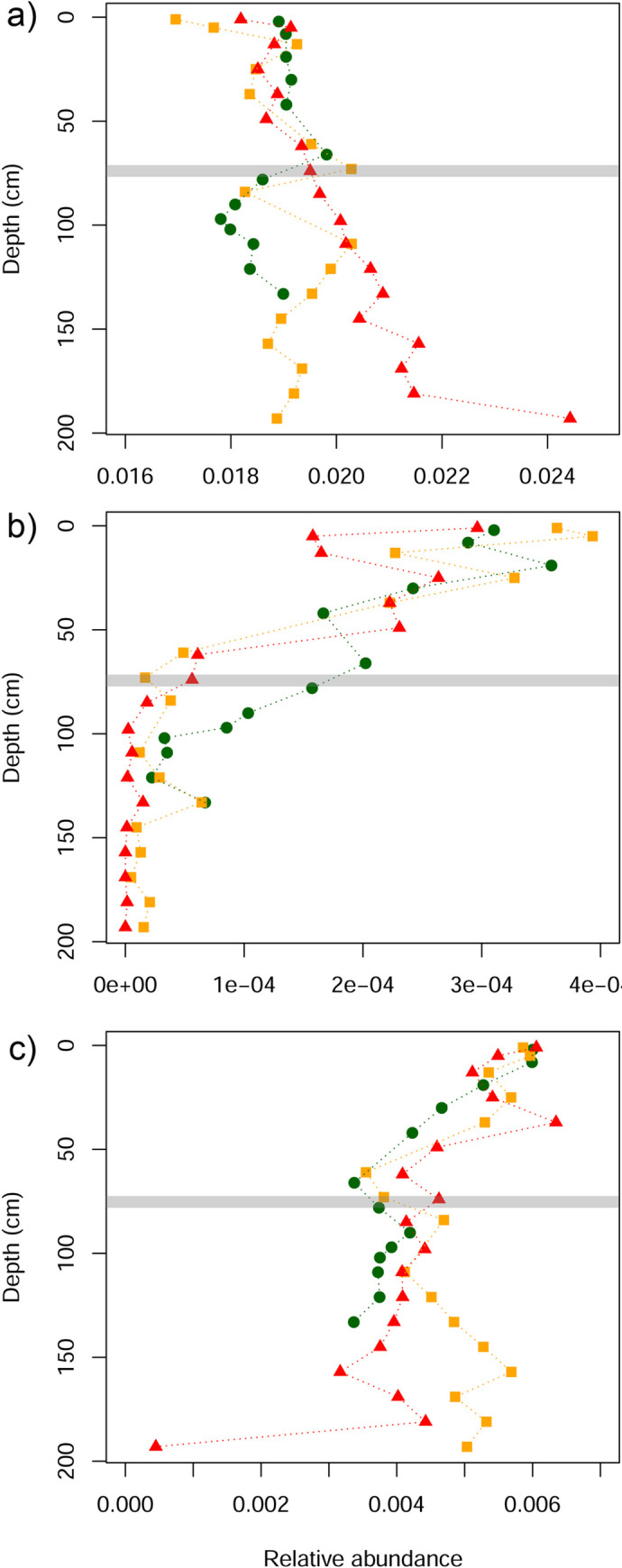
Differences in the relative abundance of metabolic pathways in three New Zealand ombrotrophic bogs along a wetland condition gradient. a) aerobic respiration; b) denitrification; c) sulfate reduction. Red, Dunearn (most modified condition); Orange, Bayswater (moderate condition); Green, Awarua (most intact condition). Horizontal line marks 75 cm depth.

**Fig 8:**
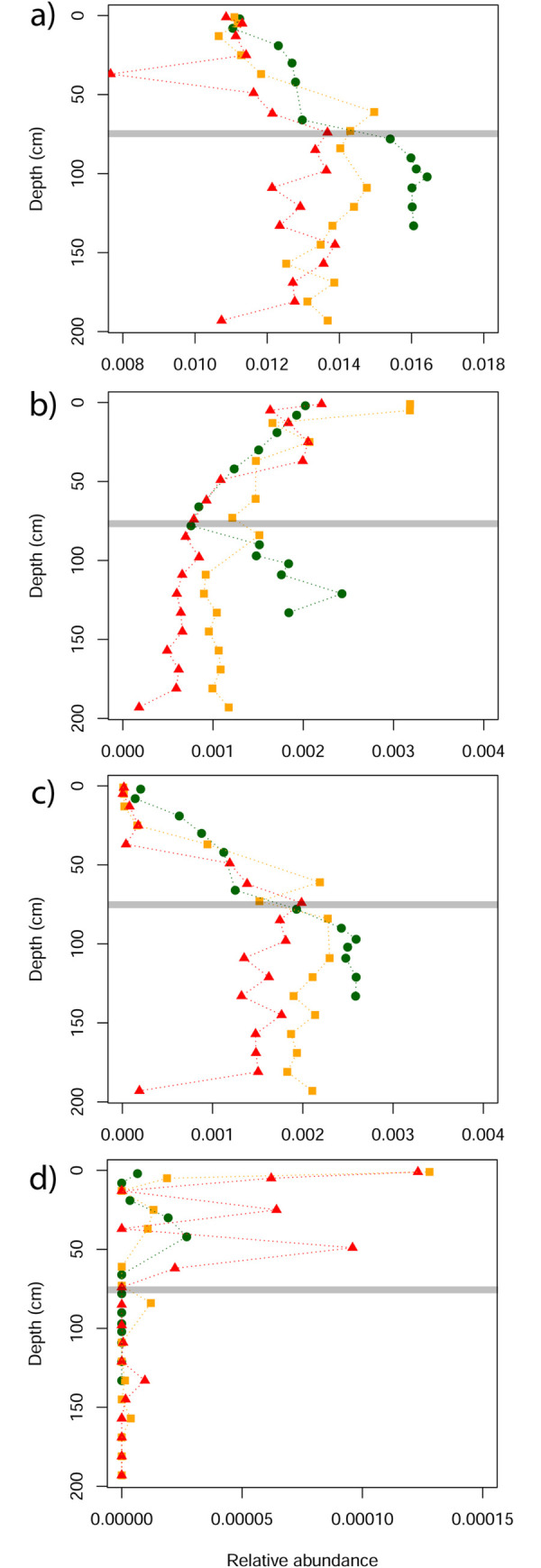
Differences in the relative abundance of metabolic pathways related to the carbon cycle in three New Zealand ombrotrophic bogs along a wetland condition gradient. a) carbon fixation; b) fermentation; c) methanogenesis; d) methanol to CO2. Red, Dunearn (most modified condition); Orange, Bayswater (moderate condition); Green, Awarua (most intact condition). Horizontal line marks 75 cm depth.

The relative abundance of selected metabolic pathways, including those contributing to key prokaryote-driven processes in wetlands, showed varying degrees of separation along the restiad ombrotrophic bog condition gradient. There were no clear patterns associated with wetland condition for denitrification or sulfate reduction pathways (Fig 7). For the aerobic respiration pathway, the three bogs had similar relative abundance values in the upper 75 cm, but below this depth the sites separated, with higher values seen in the lower condition sites (Fig 7). This pattern of site separation, either above or below ~75 cm depth, was also evident for microbial-driven processes involved in the carbon cycle. Specifically, the relative abundance of pathways contributing to carbon fixation, fermentation and methanogenesis decreased below 75 cm depth in the more degraded sites, while oxidation of methanol to CO_2_ increased in the upper 75 cm in the more degraded sites (Fig 8).

## Discussion

### Richness, diversity and community composition

Our results support a quadratic relationship between pH and prokaryote richness and diversity in New Zealand wetlands (Fig 1). Eweburn 2 and the surface peats of Glendhu and Hinekatorangihad the greatest mean richness and diversity, and had pH values ranging from 6.39–6.82. The other samples had relatively low pH (<4.5) (Awarua, Bayswater, Dunearn and Eweburn 1) or high pH (>7.39 in silts at Hinekatorangi and Glendhu) (Fig 1). The shape of the observed relationship closely matches that reported by Lauber et al. [40], who found that the highest bacterial diversity occurs in soils of pH around 6, with much lower values in soils with pH of <4.5 or >8.

Compositional patterns across New Zealand wetland sites were consistent with existing knowledge about key drivers of prokaryote community composition. For example, the increasing ratios of archaea:bacteria with depth are consistent with the findings of previous studies that have looked at a range of different soil types, including peats [41–43]. Soil pH plays a major role in structuring both archaeal [44] and bacterial communities [40]. Within our wetlands this is evident in the separation of sites in the PCA based on prokaryote communities. The pattern of separation along all three main axes formed an approximate pH gradient (from the acidic ombrotrophic bog peats through to the neutral to alkaline Hinekatorangi and Glendhu silts), with low pH sites having more positive scores (Fig 5). Patterns related to pH were also evident within both archaeal (Fig 3) and bacterial (Fig 4) communities. The high proportion of Acidobacteria in low pH wetlands (pH < 5) and increase in Firmicutes and Bacteroidetes with increasing pH were all consistent with the pH-driven patterns in soil bacterial community composition reported by Lauber et al. [40].

Interestingly, the bacterial community depth profiles of the acidic ombrotrophic bogs are consistent with those recorded from a similar site in southwestern Finland, where bacterial communities comprised mostly Acidobacteria, Proteobacteria, Veruccomicrobia and Chloroflexi [21]. This similarity demonstrates the remarkable consistency of bacterial community composition at high taxonomic ranks between wetlands of similar environmental conditions, even across large geographic distances [12].

Depth also affected prokaryote community composition, with separation of sites along PCA axis 1 occurring at between ~50-75cm depth (Fig 5). However, this was not especially evident in the plots of archaeal classes (Fig 3) or bacterial phyla (Fig 4), and so may reflect turnover at lower taxonomic ranks. This depth threshold may relate to local depths of the boundary between the upper aerobic layer (i.e. acrotelm) and the water-saturated anaerobic layer (i.e. catotelm). Although highly variable, the threshold is typically around 50cm deep in bogs [45], but can extend down to at least 70cm in New Zealand bogs with high seasonal water table variations [46].

The detection of DNA from dead cells can be a potential concern when characterising living microbial communities [47]. Although the contribution of legacy DNA to the environmental DNA (eDNA) signal retrieved from wetlands remains to be quantified it is likely to be minimal. DNA is unstable in acidic conditions such as peat bogs and degrades relatively rapidly in such environments [48, 49], and there has been limited success in retrieving old DNA from wetlands [50].

### Predicted metabolic pathways

Our results indicate an effect of both site and depth on the composition of predicted metabolic pathways (Fig 6). The top indicator pathways for each cluster can provide some insights into what might be driving this structuring (S2 Table). The ten best indicators for cluster A (which included surface samples from all wetlands) were all pathways that occur in bacteria, and also included the aerobic pathway (toluene degradation IV (aerobic) (via catechol)). This reflects the dominance of bacteria in the upper peat (Fig 2) where water-table fluctuations expose this layer to aerobic conditions. The deeper layers (>75cm) in Awarua and Bayswater both fall into cluster B. The two best indicator pathways for this cluster are found in methanogenic bacteria (*Methylobacteriaceae*) and archaea (Methanobacteria, Methanococci, Methanomicrobia) (S2 Table). By contrast, Dunearn flips between clusters B and C, the latter clusterbeing dominated by pathways found in bacteria including a couple that are specifically aerobic (purine nucleotides degradation II and heme b biosynthesis I). This pattern among the three ombrotrophic bogs appears to suggest higher rates of methanogenesis in the deeper layers at Awarua and Bayswater (Fig 8C), and higher rates of aerobic respiration in deeper layers of Dunearn, a relatively degraded site (Fig 7A). Deeper samples in Glendhu flip between clusters E and G, both of which include archaeal-specific pathways within the top ten indicators for each cluster (S2 Table). This likely reflects the higher archaea:bacteria ratios of these sediments (Fig 2).

### Effects of anthropogenic modification on wetland function

With data from just three ombrotrophic bogs it is not possible to make any definitive conclusions as to whether anthropogenic modification has consistent effects on microbial function within wetlands. However, we observed that thehe relative abundance of predicted metabolic pathways contributing to several key microbial processes exhibited patterns of differences between ombrotrophic bogs that were consistent with the condition gradient. We interpret these as all being indicators of wetland drainage, and suggest that testing the consistency of these patterns with data from additional sites would be a worthy avenue for future investigation. For example, the greater relative abundance of the aerobic respiration pathway below ~75cm depth in lower condition sites (Fig 7A) is consistent with deeper aerobic limits in drained wetlands [17]. Moreover, patterns observed in the relative abundance of metabolic pathways relating to the carbon cycle (Fig 8) also reflect the expected impacts of drainage on wetland soils. For example, the decrease in relative abundance of methanogenesis (Fig 8C) is consistent with reductions in methane (CH_4_) emissions from drained wetlands [51], while an increase in the oxidation of methanol to CO_2_ (Fig 8D) is consistent with drainage leading to increased production of CO_2_ via increased aeration of peats causing decomposition of organic matter [52]. The differences in the relative abundance of these metabolic pathways occurred down to the base of the cores (138cm in Awarua and 200cm in Bayswater and Dunearn) (Figs 7 and 8), indicating that the impacts of drainage on microbial processes may extend well below water table and deep into the water-saturated anaerobic layers. This is of concern for drying peatlands, and demonstrates that changes within microbial communities are of primary importance to CO_2_ and CH_4_ exchange.

We suggest that the observed decrease in archaea:bacteria ratios at depth in ombrotrophic bogs relative to declining wetland condition (Fig 2) may partly play a role in driving some of these observed patterns in wetland function. For example, the reduced relative abundance of methanogenic pathways in degraded bogs may reflect the fact that methanogenesis in wetlands occurs in anaerobic conditions and is driven by methanogenic archaea [53]. Conversely, an increase in methane oxidation was observed in more degraded bogs, a process that in aerobic conditions is driven by nitrifying and methane oxidising bacteria [53].

### eDNA as a tool for wetland monitoring

Effects of anthropogenic modification and protection on wetland prokaryote communities have previously been reported by studies using environmental DNA (eDNA) (e.g. [15, 54]). Our results further demonstrate that it is possible to obtain indicators of wetland condition and function from 16S rRNA metabarcoding data, and support the idea that eDNA sequencing could be developed as a useful tool for assessing the degree of wetland degradation due to anthropogenic modifications and global climate change, or monitoring wetland restoration. Importantly, our results indicate that some effects of anthropogenic modification of wetlands, especially drainage, may be expressed at depth rather than being confined to the surface of the wetland. Sampling regimes that are limited to surface soils may therefore miss some of these critical changes occurring in wetlands as a result of anthropogenic impact. The fact that, with the appropriate equipment, soil cores can readily be obtained from wetlands and eDNA can be obtained from these cores, provides an approach for assessing these changes in microbial communities and processes occurring at depth.

Although the results of our study support the idea that 16S rRNA metabarcoding can provide a useful tool for wetland assessment and monitoring, they have also opened up several avenues for further study that will help improve the potential of this tool. These aspects include: 1) identification of sensitive indicators (whether these are presence of certain prokaryote taxa, shifts in prokaryote community composition or changes in metabolic pathways) for discriminating different types of anthropogenic disturbance (e.g. drainage, eutrophication); 2) demonstrating consistency of indicator responses across different wetlands and wetland types; and 3) using temporal sampling to show whether reverse trends in indicators of anthropogenic disturbance are detectable in wetlands undergoing restoration.

## Supporting information

S1 Fig Location and stratigraphic details of wetland soil cores used in this study. Filled circles represent sample depths.
(TIF)

S2 Fig Historic and recent aerial photography showing changes in extent of three ombrotrophic bog study sites, with photographs of current vegetation at sampling sites. (TIF)

S3 Fig Assignment of samples to clusters based on the composition of predicted metabolic pathways. (TIF)

S1 Table Prokaryote taxa identified from extraction and PCR blank controls, with number of blank controls in which they were detected (n), their maximum relative abundance in a single blank control (Max %) and their average relative abundance across all blank controls (Mean %). (DOCX)

S2 Table Top predicted metabolic pathways for each cluster (maximum 10 per cluster). (DOCX)
