# Supplementary figures and images for: Vertical distribution of prokaryotes communities and predicted metabolic pathways in New Zealand wetlands, and potential for environmental DNA indicators of wetland condition
Source: PLoS One. 2021 Jan 6;16(1):e0243363. doi: 10.1371/journal.pone.0243363 (PMC7787371; doi:10.1371/journal.pone.0243363)

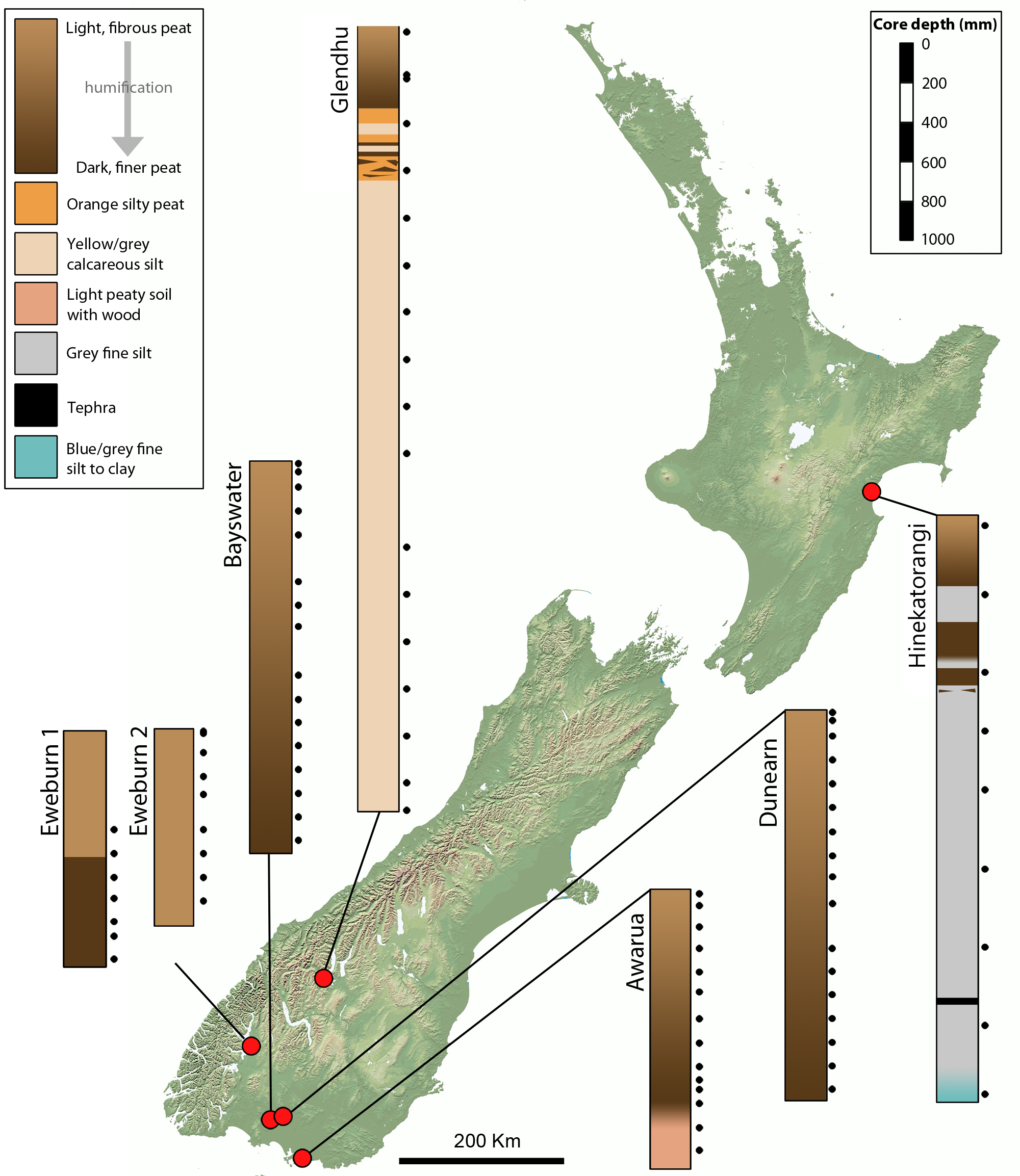

Supplement: S1 Fig — Filled circles represent sample depths. (TIF) [file pone.0243363.s001.tif]

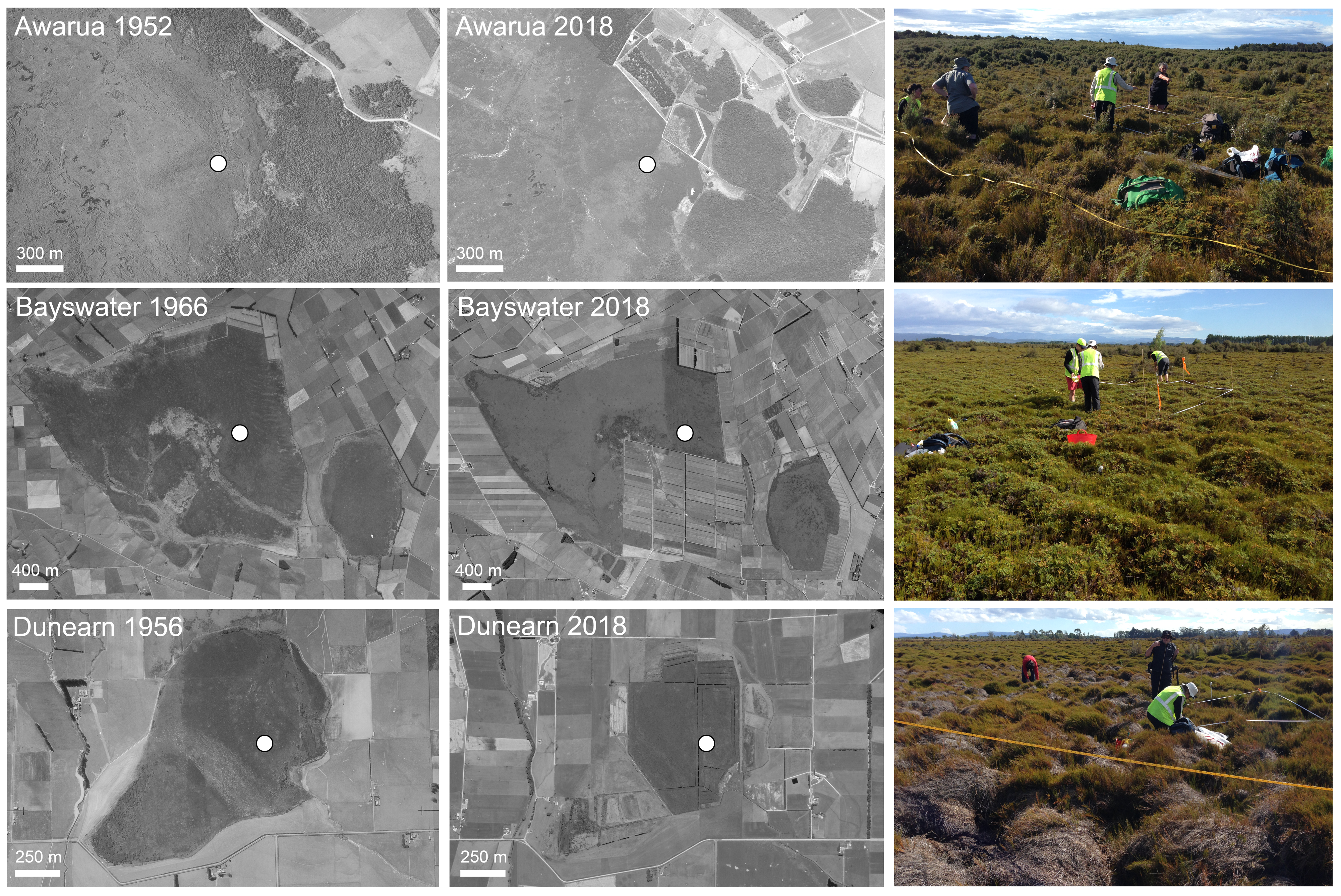

Supplement: S2 Fig — (TIF) [file pone.0243363.s002.tif]

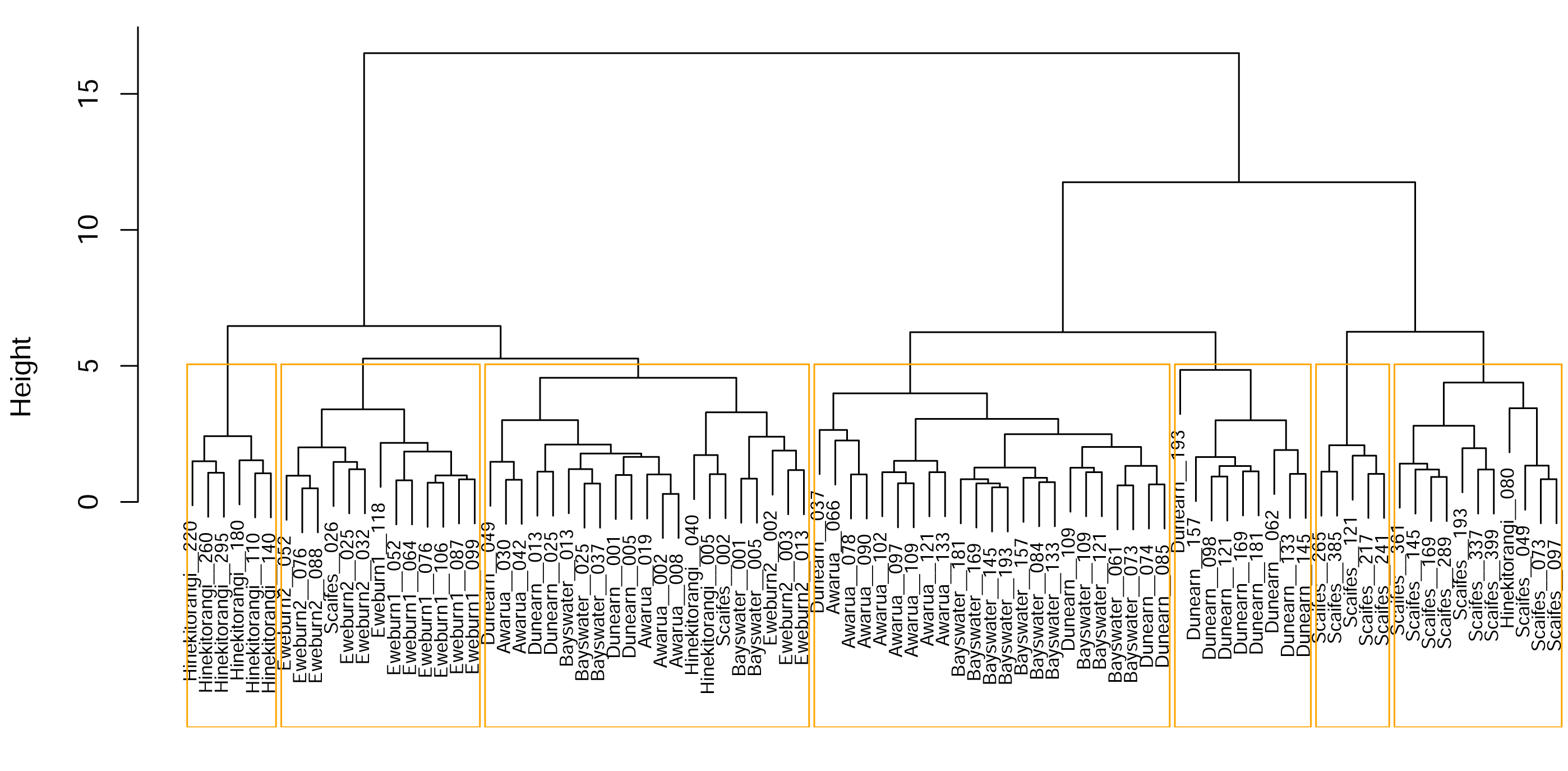

Supplement: S3 Fig — (TIF) [file pone.0243363.s003.tif]
