# Supplementary material for: Vertical distribution of prokaryotes communities and predicted metabolic pathways in New Zealand wetlands, and potential for environmental DNA indicators of wetland condition
Source: PLoS One. 2021 Jan 6;16(1):e0243363. doi: 10.1371/journal.pone.0243363 (PMC7787371; doi:10.1371/journal.pone.0243363)
Supplement: S1 Table — (DOCX) [file pone.0243363.s004.docx]

|  | **Extraction blanks** | | | **PCR blanks** | | |  |
| --- | --- | --- | --- | --- | --- | --- | --- |
| **Greengenes taxonomy** | **n** | **Max %** | **Mean %** | **n** | **Max %** | **Mean %** | **Total n blanks** |
| k__Bacteria;p__Bacteroidetes;c__Flavobacteriia;o__Flavobacteriales;f__[Weeksellaceae];g__Chryseobacterium;s__ | 8 | 25.54 | 8.53 | 3 | 25.45 | 7.43 | 11 |
| k__Bacteria;p__Proteobacteria;c__Alphaproteobacteria;o__Caulobacterales;f__Caulobacteraceae;g__Brevundimonas;s__vesicularis | 7 | 51.70 | 15.70 | 3 | 20.45 | 10.07 | 10 |
| k__Bacteria;p__Proteobacteria;c__Alphaproteobacteria;o__Rhodobacterales;f__Rhodobacteraceae;g__Paracoccus;__ | 7 | 34.99 | 12.01 | 3 | 26.01 | 14.21 | 10 |
| k__Bacteria;p__Proteobacteria;c__Alphaproteobacteria;o__Caulobacterales;f__Caulobacteraceae;g__Phenylobacterium;s__ | 7 | 66.01 | 13.58 | 2 | 42.70 | 13.17 | 9 |
| k__Bacteria;p__Proteobacteria;c__Alphaproteobacteria;o__Caulobacterales;f__Caulobacteraceae;__;__ | 5 | 2.95 | 0.48 | 3 | 2.02 | 0.73 | 8 |
| k__Bacteria;p__Proteobacteria;c__Betaproteobacteria;o__Burkholderiales;f__Comamonadaceae;__;__ | 7 | 8.91 | 2.01 | 1 | 0.24 | 0.05 | 8 |
| k__Bacteria;p__Proteobacteria;c__Deltaproteobacteria;o__Myxococcales;f__0319-6G20;g__;s__ | 5 | 15.32 | 2.92 | 3 | 14.47 | 7.27 | 8 |
| k__Bacteria;p__Cyanobacteria;c__4C0d-2;o__MLE1-12;f__;g__;s__ | 5 | 61.84 | 13.19 | 2 | 52.59 | 16.54 | 7 |
| k__Bacteria;p__Proteobacteria;c__Alphaproteobacteria;o__Rhizobiales;f__Rhizobiaceae;g__Agrobacterium;s__ | 5 | 15.52 | 1.51 | 2 | 5.63 | 1.46 | 7 |
| k__Bacteria;p__Proteobacteria;c__Alphaproteobacteria;o__Rhizobiales;f__Bradyrhizobiaceae;g__Bosea;s__genosp. | 4 | 13.34 | 1.41 | 2 | 1.84 | 0.48 | 6 |
| k__Bacteria;p__Proteobacteria;c__Alphaproteobacteria;o__Rhodobacterales;f__Rhodobacteraceae;g__Paracoccus;s__marcusii | 4 | 11.17 | 1.90 | 2 | 4.01 | 1.09 | 6 |
| k__Bacteria;p__Proteobacteria;c__Alphaproteobacteria;o__Sphingomonadales;f__Sphingomonadaceae;g__Sphingomonas;__ | 3 | 27.87 | 2.90 | 2 | 18.99 | 4.99 | 5 |
| k__Bacteria;p__Proteobacteria;c__Alphaproteobacteria;o__Sphingomonadales;f__Sphingomonadaceae;g__Sphingomonas;s__ | 4 | 2.29 | 0.36 | 1 | 0.75 | 0.15 | 5 |
| k__Bacteria;p__Proteobacteria;c__Alphaproteobacteria;o__Sphingomonadales;f__Sphingomonadaceae;g__Sphingomonas;s__echinoides | 4 | 20.93 | 3.48 | 1 | 0.01 | 0.00 | 5 |
| k__Bacteria;p__Actinobacteria;c__Actinobacteria;o__Actinomycetales;f__Micrococcaceae;g__Micrococcus;__ | 3 | 10.68 | 1.84 | 1 | 3.93 | 0.79 | 4 |
| k__Bacteria;p__Bacteroidetes;c__Flavobacteriia;o__Flavobacteriales;f__[Weeksellaceae];g__Cloacibacterium;s__ | 1 | 4.92 | 0.41 | 2 | 12.99 | 3.65 | 3 |
| k__Bacteria;p__Firmicutes;c__Bacilli;o__Bacillales;f__Staphylococcaceae;g__Staphylococcus;__ | 3 | 3.24 | 0.33 | 0 | 0.00 | 0.00 | 3 |
| k__Bacteria;p__Proteobacteria;c__Alphaproteobacteria;o__Rhodobacterales;f__Rhodobacteraceae;g__Paracoccus;s__aminovorans | 2 | 2.97 | 0.25 | 1 | 10.42 | 2.08 | 3 |
| k__Bacteria;p__Proteobacteria;c__Betaproteobacteria;o__Burkholderiales;f__Comamonadaceae;g__Comamonas;s__ | 2 | 1.70 | 0.14 | 1 | 0.01 | 0.00 | 3 |
| k__Bacteria;p__Proteobacteria;c__Betaproteobacteria;o__Rhodocyclales;f__Rhodocyclaceae;g__Hydrogenophilus;s__ | 2 | 8.84 | 1.02 | 1 | 1.43 | 0.29 | 3 |
| k__Bacteria;p__Firmicutes;c__Bacilli;o__Bacillales;f__Bacillaceae;__;__ | 1 | 0.09 | 0.01 | 1 | 0.17 | 0.03 | 2 |
| k__Bacteria;p__Firmicutes;c__Bacilli;o__Bacillales;f__Bacillaceae;g__Geobacillus;s__ | 1 | 22.09 | 1.84 | 1 | 6.27 | 1.25 | 2 |
| k__Bacteria;p__Firmicutes;c__Clostridia;o__Clostridiales;f__[Tissierellaceae];g__WAL_1855D;s__ | 2 | 9.97 | 0.87 | 0 | 0.00 | 0.00 | 2 |
| k__Bacteria;p__Proteobacteria;c__Alphaproteobacteria;o__Caulobacterales;f__Caulobacteraceae;g__Brevundimonas;s__diminuta | 2 | 1.73 | 0.19 | 0 | 0.00 | 0.00 | 2 |
| k__Bacteria;p__Proteobacteria;c__Alphaproteobacteria;o__Rhizobiales;f__Methylobacteriaceae;g__Methylobacterium;s__adhaesivum | 1 | 2.47 | 0.21 | 1 | 1.18 | 0.24 | 2 |
| k__Bacteria;p__Proteobacteria;c__Alphaproteobacteria;o__Rhodospirillales;f__Rhodospirillaceae;g__Azospirillum;s__irakense | 1 | 5.29 | 0.44 | 1 | 2.06 | 0.41 | 2 |
| k__Bacteria;p__Proteobacteria;c__Betaproteobacteria;o__Burkholderiales;f__Comamonadaceae;g__Acidovorax;__ | 1 | 0.92 | 0.08 | 1 | 1.32 | 0.26 | 2 |
| k__Bacteria;p__Proteobacteria;c__Deltaproteobacteria;o__Bdellovibrionales;f__Bacteriovoracaceae;g__;s__ | 1 | 1.62 | 0.13 | 1 | 1.41 | 0.28 | 2 |
| k__Bacteria;p__Proteobacteria;c__Gammaproteobacteria;o__Pseudomonadales;f__Moraxellaceae;g__Acinetobacter;__ | 1 | 8.96 | 0.75 | 1 | 1.81 | 0.36 | 2 |
| k__Bacteria;p__Proteobacteria;c__Gammaproteobacteria;o__Pseudomonadales;f__Moraxellaceae;g__Acinetobacter;s__ | 1 | 0.02 | 0.00 | 1 | 2.59 | 0.52 | 2 |
| k__Bacteria;p__Proteobacteria;c__Gammaproteobacteria;o__Pseudomonadales;f__Moraxellaceae;g__Enhydrobacter;s__ | 2 | 3.23 | 0.43 | 0 | 0.00 | 0.00 | 2 |
| k__Bacteria;p__Proteobacteria;c__Gammaproteobacteria;o__Pseudomonadales;f__Pseudomonadaceae;g__Pseudomonas;s__veronii | 2 | 6.15 | 0.58 | 0 | 0.00 | 0.00 | 2 |
| k__Bacteria;p__Proteobacteria;c__Gammaproteobacteria;o__Xanthomonadales;f__Sinobacteraceae;g__;s__ | 2 | 0.01 | 0.00 | 0 | 0.00 | 0.00 | 2 |
| k__Bacteria;p__Proteobacteria;c__Gammaproteobacteria;o__Xanthomonadales;f__Xanthomonadaceae;g__Luteimonas;s__ | 2 | 3.12 | 0.43 | 0 | 0.00 | 0.00 | 2 |
| k__Bacteria;__;__;__;__;__;__ | 1 | 0.00 | 0.00 | 0 | 0.00 | 0.00 | 1 |
| k__Bacteria;p__;c__;o__;f__;g__;s__ | 1 | 0.00 | 0.00 | 0 | 0.00 | 0.00 | 1 |
| k__Bacteria;p__Acidobacteria;c__DA052;o__Ellin6513;f__;g__;s__ | 1 | 0.02 | 0.00 | 0 | 0.00 | 0.00 | 1 |
| k__Bacteria;p__Acidobacteria;c__Solibacteres;o__Solibacterales;__;__;__ | 1 | 0.01 | 0.00 | 0 | 0.00 | 0.00 | 1 |
| k__Bacteria;p__Acidobacteria;c__Solibacteres;o__Solibacterales;f__;g__;s__ | 1 | 0.01 | 0.00 | 0 | 0.00 | 0.00 | 1 |
| k__Bacteria;p__Acidobacteria;c__TM1;o__;f__;g__;s__ | 1 | 0.01 | 0.00 | 0 | 0.00 | 0.00 | 1 |
| k__Bacteria;p__Actinobacteria;c__Actinobacteria;o__Actinomycetales;f__Brevibacteriaceae;g__Brevibacterium;__ | 1 | 6.93 | 0.58 | 0 | 0.00 | 0.00 | 1 |
| k__Bacteria;p__Actinobacteria;c__Actinobacteria;o__Actinomycetales;f__Corynebacteriaceae;g__Corynebacterium;s__simulans | 0 | 0.00 | 0.00 | 1 | 7.86 | 1.57 | 1 |
| k__Bacteria;p__Actinobacteria;c__Actinobacteria;o__Actinomycetales;f__Dermabacteraceae;g__Brachybacterium;__ | 0 | 0.00 | 0.00 | 1 | 1.30 | 0.26 | 1 |
| k__Bacteria;p__Actinobacteria;c__Actinobacteria;o__Actinomycetales;f__Micrococcaceae;g__Micrococcus;s__ | 1 | 0.07 | 0.01 | 0 | 0.00 | 0.00 | 1 |
| k__Bacteria;p__Actinobacteria;c__Actinobacteria;o__Actinomycetales;f__Micrococcaceae;g__Micrococcus;s__luteus | 1 | 1.58 | 0.13 | 0 | 0.00 | 0.00 | 1 |
| k__Bacteria;p__Bacteroidetes;__;__;__;__;__ | 0 | 0.00 | 0.00 | 1 | 0.01 | 0.00 | 1 |
| k__Bacteria;p__Bacteroidetes;c__Cytophagia;o__Cytophagales;f__Cytophagaceae;g__Cytophaga;s__ | 1 | 1.38 | 0.11 | 0 | 0.00 | 0.00 | 1 |
| k__Bacteria;p__Bacteroidetes;c__Sphingobacteriia;o__Sphingobacteriales;f__Sphingobacteriaceae;g__;s__ | 1 | 0.19 | 0.02 | 0 | 0.00 | 0.00 | 1 |
| k__Bacteria;p__Bacteroidetes;c__Sphingobacteriia;o__Sphingobacteriales;f__Sphingobacteriaceae;g__Sphingobacterium;s__multivorum | 1 | 0.63 | 0.05 | 0 | 0.00 | 0.00 | 1 |
| k__Bacteria;p__Bacteroidetes;c__[Saprospirae];o__[Saprospirales];f__Chitinophagaceae;g__Sediminibacterium;s__ | 0 | 0.00 | 0.00 | 1 | 5.88 | 1.18 | 1 |
| k__Bacteria;p__Chlamydiae;c__Chlamydiia;o__Chlamydiales;f__Rhabdochlamydiaceae;g__Candidatus Rhabdochlamydia;s__ | 1 | 0.00 | 0.00 | 0 | 0.00 | 0.00 | 1 |
| k__Bacteria;p__Firmicutes;c__Bacilli;o__Bacillales;__;__;__ | 1 | 0.36 | 0.03 | 0 | 0.00 | 0.00 | 1 |
| k__Bacteria;p__Firmicutes;c__Bacilli;o__Bacillales;f__Bacillaceae;g__Bacillus;s__flexus | 1 | 79.95 | 6.66 | 0 | 0.00 | 0.00 | 1 |
| k__Bacteria;p__Firmicutes;c__Bacilli;o__Bacillales;f__Bacillaceae;g__Natronobacillus;s__ | 0 | 0.00 | 0.00 | 1 | 7.93 | 1.59 | 1 |
| k__Bacteria;p__OD1;c__Mb-NB09;o__;f__;g__;s__ | 1 | 1.73 | 0.14 | 0 | 0.00 | 0.00 | 1 |
| k__Bacteria;p__Proteobacteria;c__Alphaproteobacteria;o__Caulobacterales;f__Caulobacteraceae;g__;s__ | 1 | 0.15 | 0.01 | 0 | 0.00 | 0.00 | 1 |
| k__Bacteria;p__Proteobacteria;c__Alphaproteobacteria;o__Caulobacterales;f__Caulobacteraceae;g__Brevundimonas;__ | 1 | 0.02 | 0.00 | 0 | 0.00 | 0.00 | 1 |
| k__Bacteria;p__Proteobacteria;c__Alphaproteobacteria;o__Caulobacterales;f__Caulobacteraceae;g__Brevundimonas;s__ | 0 | 0.00 | 0.00 | 1 | 6.67 | 1.33 | 1 |
| k__Bacteria;p__Proteobacteria;c__Alphaproteobacteria;o__Rhizobiales;f__Bradyrhizobiaceae;g__Balneimonas;s__ | 0 | 0.00 | 0.00 | 1 | 2.12 | 0.42 | 1 |
| k__Bacteria;p__Proteobacteria;c__Alphaproteobacteria;o__Rhizobiales;f__Bradyrhizobiaceae;g__Bradyrhizobium;__ | 1 | 0.10 | 0.01 | 0 | 0.00 | 0.00 | 1 |
| k__Bacteria;p__Proteobacteria;c__Alphaproteobacteria;o__Rhizobiales;f__Bradyrhizobiaceae;g__Bradyrhizobium;s__ | 1 | 1.25 | 0.10 | 0 | 0.00 | 0.00 | 1 |
| k__Bacteria;p__Proteobacteria;c__Alphaproteobacteria;o__Rhizobiales;f__Rhizobiaceae;__;__ | 0 | 0.00 | 0.00 | 1 | 1.08 | 0.22 | 1 |
| k__Bacteria;p__Proteobacteria;c__Alphaproteobacteria;o__Rhodobacterales;f__Rhodobacteraceae;g__Paracoccus;s__ | 0 | 0.00 | 0.00 | 1 | 0.39 | 0.08 | 1 |
| k__Bacteria;p__Proteobacteria;c__Alphaproteobacteria;o__Rhodospirillales;f__Rhodospirillaceae;g__Azospirillum;s__ | 1 | 0.08 | 0.01 | 0 | 0.00 | 0.00 | 1 |
| k__Bacteria;p__Proteobacteria;c__Alphaproteobacteria;o__Rhodospirillales;f__Rhodospirillaceae;g__Novispirillum;s__ | 0 | 0.00 | 0.00 | 1 | 1.02 | 0.20 | 1 |
| k__Bacteria;p__Proteobacteria;c__Alphaproteobacteria;o__Rhodospirillales;f__Rhodospirillaceae;g__Reyranella;s__massiliensis | 1 | 5.25 | 0.44 | 0 | 0.00 | 0.00 | 1 |
| k__Bacteria;p__Proteobacteria;c__Alphaproteobacteria;o__Rhodospirillales;f__Rhodospirillaceae;g__Telmatospirillum;s__ | 1 | 0.00 | 0.00 | 0 | 0.00 | 0.00 | 1 |
| k__Bacteria;p__Proteobacteria;c__Alphaproteobacteria;o__Sphingomonadales;f__Sphingomonadaceae;g__Novosphingobium;__ | 0 | 0.00 | 0.00 | 1 | 3.48 | 0.70 | 1 |
| k__Bacteria;p__Proteobacteria;c__Betaproteobacteria;o__Burkholderiales;f__Oxalobacteraceae;g__Janthinobacterium;s__lividum | 1 | 5.50 | 0.46 | 0 | 0.00 | 0.00 | 1 |
| k__Bacteria;p__Proteobacteria;c__Betaproteobacteria;o__Burkholderiales;f__Oxalobacteraceae;g__Ralstonia;s__ | 0 | 0.00 | 0.00 | 1 | 0.06 | 0.01 | 1 |
| k__Bacteria;p__Proteobacteria;c__Betaproteobacteria;o__Rhodocyclales;f__Rhodocyclaceae;g__Uliginosibacterium;s__ | 0 | 0.00 | 0.00 | 1 | 6.08 | 1.22 | 1 |
| k__Bacteria;p__Proteobacteria;c__Gammaproteobacteria;o__Aeromonadales;f__Aeromonadaceae;__;__ | 1 | 2.18 | 0.18 | 0 | 0.00 | 0.00 | 1 |
| k__Bacteria;p__Proteobacteria;c__Gammaproteobacteria;o__Pasteurellales;f__Pasteurellaceae;g__Haemophilus;s__parainfluenzae | 1 | 0.27 | 0.02 | 0 | 0.00 | 0.00 | 1 |
| k__Bacteria;p__Proteobacteria;c__Gammaproteobacteria;o__Pseudomonadales;f__Moraxellaceae;g__Acinetobacter;s__lwoffii | 1 | 6.01 | 0.50 | 0 | 0.00 | 0.00 | 1 |
| k__Bacteria;p__Proteobacteria;c__Gammaproteobacteria;o__Pseudomonadales;f__Moraxellaceae;g__Enhydrobacter;__ | 0 | 0.00 | 0.00 | 1 | 3.49 | 0.70 | 1 |
| k__Bacteria;p__Proteobacteria;c__Gammaproteobacteria;o__Pseudomonadales;f__Pseudomonadaceae;g__;s__ | 0 | 0.00 | 0.00 | 1 | 6.49 | 1.30 | 1 |
| k__Bacteria;p__Proteobacteria;c__Gammaproteobacteria;o__Pseudomonadales;f__Pseudomonadaceae;g__Pseudomonas;__ | 1 | 7.07 | 0.59 | 0 | 0.00 | 0.00 | 1 |
| k__Bacteria;p__Proteobacteria;c__Gammaproteobacteria;o__Pseudomonadales;f__Pseudomonadaceae;g__Pseudomonas;s__ | 0 | 0.00 | 0.00 | 1 | 4.88 | 0.98 | 1 |
| k__Bacteria;p__Proteobacteria;c__Gammaproteobacteria;o__Xanthomonadales;f__Xanthomonadaceae;g__Pseudoxanthomonas;__ | 0 | 0.00 | 0.00 | 1 | 2.29 | 0.46 | 1 |
| k__Bacteria;p__Verrucomicrobia;c__[Pedosphaerae];o__[Pedosphaerales];f__;g__;s__ | 1 | 0.01 | 0.00 | 0 | 0.00 | 0.00 | 1 |
| k__Bacteria;p__Verrucomicrobia;c__[Pedosphaerae];o__[Pedosphaerales];f__[Pedosphaeraceae];g__Pedosphaera;s__ | 1 | 0.00 | 0.00 | 0 | 0.00 | 0.00 | 1 |
| k__Bacteria;p__Verrucomicrobia;c__[Pedosphaerae];o__[Pedosphaerales];f__auto67_4W;g__;s__ | 1 | 0.00 | 0.00 | 0 | 0.00 | 0.00 | 1 |
