# Supplementary material for: Vertical distribution of prokaryotes communities and predicted metabolic pathways in New Zealand wetlands, and potential for environmental DNA indicators of wetland condition
Source: PLoS One. 2021 Jan 6;16(1):e0243363. doi: 10.1371/journal.pone.0243363 (PMC7787371; doi:10.1371/journal.pone.0243363)
Supplement: S2 Table — (DOCX) [file pone.0243363.s005.docx]

| **Cluster** | **Metacyc code** | **Pathway description** | **Indicator value** | **p** | **Expected taxonomic range (among archaea and bacteria)** |
| --- | --- | --- | --- | --- | --- |
| A | PWY-7255 | ergothioneine biosynthesis I (bacteria) | 0.8346 | 0.001* | Bacteria  Actinobacteria, Alphaproteobacteria, Bacteroidetes, Betaproteobacteria, Cyanobacteria, Deltaproteobacteria, Gammaproteobacteria |
|  | P163-PWY | L-lysine fermentation to acetate and butanoate | 0.7315 | 0.001* | Bacteria |
|  | PWY-5178 | toluene degradation IV (aerobic) (via catechol) | 0.6883 | 0.001* | Bacteria  Proteobacteria |
|  | PWY-3661 | glycine betaine degradation I | 0.6765 | 0.001* | Archaea  Bacteria |
|  | PWY-6383 | mono-trans, poly-cis decaprenyl phosphate biosynthesis | 0.6729 | 0.001* | Bacteria  Mycobacteriaceae |
|  | PWY-5392 | reductive TCA cycle II (carbon fixation) | 0.6722 | 0.001* | Bacteria  Aquificae |
|  | HCAMHPDEG-PWY | 3-phenylpropanoate and 3-(3-hydroxyphenyl)propanoate degradation to 2-hydroxypentadienoate | 0.6685 | 0.001* | Bacteria  Proteobacteria |
|  | PWY-6690 | cinnamate and 3-hydroxycinnamate degradation to 2-hydroxypentadienoate | 0.6685 | 0.001* | Bacteria |
|  | PWY-7616 | methanol oxidation to carbon dioxide | 0.6644 | 0.001* | Bacteria |
|  | PWY-6397 | mycolyl-arabinogalactan-peptidoglycan complex biosynthesis | 0.6245 | 0.001* | Bacteria  Mycobacteriaceae |
| B | PWY-6148 | tetrahydromethanopterin biosynthesis | 0.3773 | 0.002* | Bacteria  Methanobacteria, Methanococci, Methanomicrobia, Methylobacteriaceae |
|  | P241-PWY | coenzyme B biosynthesis | 0.3431 | 0.014* | Bacteria  Methanobacteria, Methanococci, Methanomicrobia, Methanopyri |
|  | PWY-5656 | mannosylglycerate biosynthesis I | 0.3164 | 0.014* | Archaea  Bacteria |
|  | PWY-722 | nicotinate degradation I | 0.2969 | 0.031* | Bacteria  Proteobacteria |
|  | P4-PWY | superpathway of L-lysine, L-threonine and L-methionine biosynthesis I | 0.2082 | 0.001* | Bacteria |
|  | PWY0-781 | aspartate superpathway | 0.2069 | 0.001* | Bacteria |
|  | PWY-7200 | superpathway of pyrimidine deoxyribonucleoside salvage | 0.1613 | 0.043* | Archaea  Bacteria |
|  | PWY-7385 | 1,3-propanediol biosynthesis | 0.0870 | 0.383 | Engineered |
| C | PWY-6478 | GDP-D-glycero-α-D-manno-heptose biosynthesis | 0.3587 | 0.001* | Bacteria |
|  | PWY-7374 | 1,4-dihydroxy-6-naphthoate biosynthesis I | 0.3569 | 0.001* | Bacteria |
|  | PWY-4984 | urea cycle | 0.2980 | 0.001* | Bacteria |
|  | PWY-7371 | 1,4-dihydroxy-6-naphthoate biosynthesis II | 0.2927 | 0.001* | Bacteria |
|  | GLYOXYLATE-BYPASS | glyoxylate cycle | 0.2737 | 0.001* | Archaea  Bacteria |
|  | PWY-6353 | purine nucleotides degradation II (aerobic) | 0.2607 | 0.001* | Archaea  Bacteria |
|  | HEME-BIOSYNTHESIS-II | heme b biosynthesis I (aerobic) | 0.2512 | 0.001* | Bacteria |
|  | HISDEG-PWY | L-histidine degradation | 0.2436 | 0.001* | Bacteria |
|  | SALVADEHYPOX-PWY | adenosine nucleotides degradation II | 0.2425 | 0.001* | Archaea  Bacteria |
|  | HOMOSER-METSYN-PWY | L-methionine biosynthesis I | 0.2338 | 0.001* | Bacteria |
| D | PWY-6713 | L-rhamnose degradation II | 0.7987 | 0.001* | Bacteria |
|  | PWY-7046 | 4-coumarate degradation (anaerobic) | 0.5275 | 0.002* | Bacteria |
|  | ARGDEG-PWY | superpathway of L-arginine, putrescine, and 4-aminobutanoate degradation | 0.5244 | 0.001* | Bacteria |
|  | ORNARGDEG-PWY | superpathway of L-arginine and L-ornithine degradation | 0.5244 | 0.001* | Bacteria |
|  | PWY0-41 | allantoin degradation IV (anaerobic) | 0.5201 | 0.002* | Bacteria |
|  | LPSSYN-PWY | superpathway of lipopolysaccharide biosynthesis | 0.4937 | 0.003* | Bacteria |
|  | METHGLYUT-PWY | superpathway of methylglyoxal degradation | 0.4927 | 0.003* | Bacteria |
|  | GOLPDLCAT-PWY | superpathway of glycerol degradation to 1,3-propanediol | 0.4905 | 0.001* | Bacteria  Firmicutes, Proteobacteria |
|  | PWY-6629 | superpathway of L-tryptophan biosynthesis | 0.4700 | 0.005* | Bacteria |
|  | PWY-5677 | succinate fermentation to butanoate | 0.4633 | 0.023* | Bacteria  Firmicutes |
| E | PWY-6174 | mevalonate pathway II (archaea) | 0.4421 | 0.001* | Archaea |
|  | PWY-7391 | isoprene biosynthesis II | 0.3695 | 0.009* | Engineered |
|  | PWY-6167 | flavin biosynthesis II (archaea) | 0.3691 | 0.004* | Archaea |
|  | PWY-922 | mevalonate pathway I | 0.3648 | 0.002* | Archaea  Bacteria |
|  | PWY-5910 | superpathway of geranylgeranyldiphosphate biosynthesis I (via mevalonate) | 0.3627 | 0.002* | Bacteria |
|  | PWY-5198 | factor 420 biosynthesis | 0.3333 | 0.004* | Archaea  Bacteria  Actinobacteria, Cyanobacteria |
|  | PWY-5005 | biotin biosynthesis II | 0.3240 | 0.019* | Bacteria |
|  | PWY-6895 | superpathway of thiamine diphosphate biosynthesis II | 0.3219 | 0.071 | Bacteria |
|  | PWY-5532 | nucleoside and nucleotide degradation (archaea) | 0.3113 | 0.028* | Archaea |
|  | P162-PWY | L-glutamate degradation V (via hydroxyglutarate) | 0.2996 | 0.082 | Bacteria  Firmicutes, Fusobacteria |
| F | PWY-6143 | CMP-pseudaminate biosynthesis | 0.9664 | 0.001* | Bacteria |
|  | PWY-4361 | S-methyl-5-thio-α-D-ribose 1-phosphate degradation I | 0.9178 | 0.001* | Archaea  Bacteria |
|  | PWY-6505 | L-tryptophan degradation XII (*Geobacillus*) | 0.9142 | 0.001* | Bacteria |
|  | PWY-7003 | glycerol degradation to butanol | 0.8947 | 0.001* | Bacteria |
|  | PWY-7527 | L-methionine salvage cycle III | 0.8884 | 0.001* | Archaea  Bacteria |
|  | PWY-7373 | superpathway of demethylmenaquinol-6 biosynthesis II | 0.8816 | 0.001* | Bacteria |
|  | P125-PWY | superpathway of (R,R)-butanediol biosynthesis | 0.8735 | 0.001* | Bacteria |
|  | GALLATE-DEGRADATION-I-PWY | gallate degradation II | 0.8675 | 0.001* | Bacteria |
|  | PWY-2941 | L-lysine biosynthesis II | 0.8591 | 0.001* | Bacteria  Firmicutes |
|  | PWY-5647 | 2-nitrobenzoate degradation I | 0.8428 | 0.001* | Bacteria |
| G | PWY-7286 | 7-(3-amino-3-carboxypropyl)-wyosine biosynthesis | 0.5361 | 0.002* | Archaea  Euryarchaeota |
|  | PWY-5177 | glutaryl-CoA degradation | 0.4353 | 0.008* | Bacteria |
|  | PWY-6349 | CDP-archaeol biosynthesis | 0.4137 | 0.002* | Archaea |
|  | PWY-6350 | archaetidylinositol biosynthesis | 0.4093 | 0.001* | Archaea |
|  | CENTBENZCOA-PWY | benzoyl-CoA degradation II (anaerobic) | 0.3681 | 0.016* | Bacteria |
|  | PWY-1861 | formaldehyde assimilation II (assimilatory RuMP Cycle) | 0.3114 | 0.001* | Bacteria |
|  | CENTFERM-PWY | pyruvate fermentation to butanoate | 0.3077 | 0.008* | Bacteria  Firmicutes, Proteobacteria |
|  | PWY-6590 | superpathway of *Clostridium acetobutylicum* acidogenic fermentation | 0.3056 | 0.007* | Bacteria  Firmicutes |
|  | PWY-7210 | pyrimidine deoxyribonucleotides biosynthesis from CTP | 0.2896 | 0.001* | Bacteria  Actinobacteria, Firmicutes |
|  | PWY-6901 | superpathway of glucose and xylose degradation | 0.2785 | 0.008* | Bacteria |
